# Non-high-density lipoprotein cholesterol/high-density lipoprotein cholesterol ratio serve as a predictor for coronary collateral circulation in chronic total occlusive patients

**DOI:** 10.1186/s12872-021-02129-9

**Published:** 2021-06-23

**Authors:** Ya Li, Xin Chen, Shu Li, Yulin Ma, Jialing Li, Mingying Lin, Jing Wan

**Affiliations:** 1 Department of Cardiology, Zhongnan Hospital of Wuhan University, No 169 Donghu Road, Wuchang District, Wuhan, 430071 Hubei Province China; 2 Department of Cardiology, The Central Hospital of Enshi Tujia and Miao Autonomous Prefecture, Enshi, 445000 China; 3 Department of Cardiology, Hubei Jianghan Oilfield General Hospital, Qianjiang, 433100 China

**Keywords:** Non-HDL-C/HDL, Coronary collateral circulation, Chronic total occlusive disease

## Abstract

**Objective:**

The present study investigated the potential correlation between non-high-density lipoprotein cholesterol/high-density lipoprotein cholesterol ratio (non-HDL-C/HDL) and the formation of coronary collateral circulation (CCC) in coronary artery disease cases with chronic total occlusive (CTO) lesions.

**Methods:**

Two experienced cardiologists identified and selected patients with CTO lesions for retrospective analysis. The 353 patients were divided into a CCC poor formation group (Rentrop 0–1 grade, n = 209) and a CCC good formation group (Rentrop 2–3 grade, n = 144) based on the Cohen-Rentrop standard. A comparison of non-HDL-C/HDL ratios between the two groups was performed. The Spearman test was used to obtain the correlation between the cholesterol ratio and Rentrop grade. Independent predictors of CCC were analyzed using logistic regression. Receiver operating characteristic (ROC) curve analysis was also performed to quantify the predictive value of research indicator.

**Results:**

The non-HDL-C/HDL ratio in the CCC poor formation group was elevated markedly compared to the CCC good formation group [( 3.86 ± 1.40) vs ( 3.31 ± 1.22), *P* = 0.000]. The Spearman test results indicated that non-HDL-C/HDL negatively correlated with Rentrop grade (r = − 0.115, *P* = 0.030). Multivariate logistic regression analysis showed that non-HDL-C/HDL ratio was an independent predictor of CCC formation (OR = 1.195, 95%CI = 1.020–1.400, *P* = 0.027). The area under the curve of ROC for detecting CCC poor formation was 0.611 (95% CI: 0.551–0.671, *P* = 0.000) with an optimal cut-off value of 2.77.

**Conclusion:**

Non-HDL-C/HDL negatively correlated with the formation of CCC and served as an independent predictor of CCC formation, which may be used as a biomarker for the evaluation of CCC.

## Introduction

Chronic total occlusions (CTOs) are an important subtype of coronary artery disease (CAD) and pose a great threat to a patient’s prognosis because it remains prevalent in approximately one third of cases with CAD [1, 2]. CTO refers to a lesion in which the coronary artery is completely occluded or almost completely occluded for more than 3 months [3]. A system of coronary collateral circulation (CCC) is found in most CTO patients. These vessels provide the myocardium with another blood supply that may prevent myocardial necrosis and help improve left ventricular function [4, 5]. Previous studies suggested that well-formed CCC in patients with CTO reduced myocardial infarcts and improved the prognosis of patients [6, 7]. Therefore, it will be worthwhile to understand the mechanisms and influencing factors of the formation of CCC to identify new biomarkers. The existing evaluation methods (e.g., Collateral Flow Index (CFI), contrast echocardiography, and myocardial perfusion imaging) for the formation of CCC in patients with CTO are relatively complicated and expensive. Thus, an easy and feasible method for evaluating or predicting CCC formation is needed.

Dyslipidemia is tightly related to atherosclerosis. In clinical practice, decrease concentration of low-density lipoprotein cholesterol (LDL-C), non-high-density lipoprotein cholesterol (non-HDL-C), and triglyceride (TG) and increase of high-density lipoprotein cholesterol (HDL-C) concentration are commonly used as parameters in lipid-lowering strategies. Clinical physicians examined the non-HDL-C/HDL, this index represents the ratio of cholesterol contained in all of the atherosclerosis-promoting particles (non-HDL-C) and cholesterol contained in all the anti-atherosclerosis particles (HDL-C) in the serum, to evaluate lipid-related diseases. Some findings indicated that this cholesterol ratio was a better index than apolipoprotein B/apolipoprotein A1 and conventional single lipid parameter for identifying lipid-related diseases, such as insulin resistance, metabolic syndrome and CAD [8, 9]. However, few studies focused on the role of non-HDL-C/HDL ratio for predicting CCC formation in patients with CTO. The present study estimated the association between the non-HDL-C/HDL ratio and the formation of CCC.

## Patients and methods

### Study population

Coronary angiography was performed in the Department of Cardiology, Zhongnan Hospital of Wuhan University from January 2013 to September 2018, and 353 patients who met the criteria were enrolled in this study. CTO diagnostic criteria were according to the American College of Cardiology Foundation (ACCF)/American Heart Association (AHA)/Society for Cardiovascular Angiography and Interventions (SCAI) of 2013[10]: the coronary vascular cavity was completely occluded because of thrombosis and the repeated organization of coronary arteries because of atherosclerotic lesions, and the course of the occlusion exceeded three months. Coronary angiography showed that three main coronary arteries (left anterior descending artery (LAD), left circumflex artery (LCA) and right coronary artery (RCA)) had at least one branch with stenosis ≥ 90%.

Exclusion criteria: Acute myocardial infarction in the past three months, previous coronary stent placement or Coronary artery bypass graft within three months, coronary artery myocardial bridge and/or congenital coronary arterial malformation, cardiomyopathy or decompensated cardiac dysfunction or other severe medical disease (liver and kidney disease, infectious diseases and thyroid disease) and patients with long-term lipid-affecting drug use history. The Medical Ethics Committee of Zhongnan Hospital approved this study.

### Assessment of coronary collateral circulation and group

Two interventional experts performed the coronary angiography based on the Judkin method using a radial or femoral approach. The degree of stenosis of main coronary arteries (LAD, LCX or RCA) was evaluated. Multivessel lesion is generally defined by the presence of a ⩾50% stenotic lesion (by visual angiographic assessment) in two or more major epicardial coronary arteries (LAD, LCX or RCA). CCC formation was evaluated using the Cohen-Rentrop criteria [11]: Grade 0, without any collateral artery filling; Grade 1, filling of the side branches of the occluded artery, and contrast agent cannot reach the epicardial vessel; Grade 2, the epicardial vessel is partially filled; and Grade 3, the epicardial vessel is completely filled by collaterals. The participants were divided into a well-formed CCC group (Rentrop grade 2–3) and a poorly formed CCC group (Rentrop grade 0–1).

### Laboratory measurements

The patients fasted for more than 10 h before blood was collected from the antecubital vein. The serum was collected after the blood has spontaneously coagulated and been centrifuged, measurement of serum lipids was performed using an automated biochemical analyzer (Beckman Coulter, AU5800, USA) and an enzymatic reaction method. Non-HDL-C equals serum total cholesterol (TC) minus serum HDL. Non-HDL-C/HDL was calculated by dividing the non-HDL-C with HDL.

### Statistical analysis

The Kolmogorov–Smirnov test was used to identify whether the measurement data were normally distributed. The continuous data in a normal distribution are represented as the means ± SD. Student’s t-test was used to identify differences between two groups. Nonnormally distributed continuous variables are represented as medians (interquartile ranges), and the difference between groups was analyzed using the Mann–Whitney U-test. The counted variables are expressed in percentages and compared using the χ^2^-test. One-way ANOVA was performed to compare the categories of Rentrop grades. The Spearman test was performed to describe correlations of research indicators with the Rentrop grade. To identify the affecting factors of CCC formation, a regressed model of multivariate logistic was used. A receiver operating characteristic (ROC) curve was analyzed to assess the research indicator for predicting CCC poor formation. The results are expressed within 95% confidence intervals (CI), and a *P* < 0.05 represented statistical significance. All the statistical variables were obtained using IBM SPSS 23.0 software (IBM Corp, Armonk, New York, USA).

## Results

### Basic clinical information and characteristics of patients

According to the evaluation of CCC formation, 144 cases had well-formed CCC, and 209 cases had poorly formed CCC. Table 1 shows that the CCC good formation group had a higher percentage of multiple vessel lesions and HDL-C was elevated in CCC good formation group. For the CCC poor formation group, the percentages of left circumflex artery occlusion and non-HDL-C/HDL [( 3.86 ± 1.40) vs ( 3.31 ± 1.22), *P* = 0.000]were elevated compared to the CCC good formation group.

**Table 1:** Basic clinical characteristics of the study participants

Variables	CCC good formation group (n = 144)	CCC poor formation group (n = 209)	*P* value
Age	63.27 ± 10.78	61.52 ± 12.16	0.165
Gender [man (%)]	122 (41.9)	169 (58.1)	0.349
Smoking	69 (41.3)	98 (58.7)	0.914
Diabetes	38 (38.0)	62 (62.0)	0.549
Hypertension	95 (43.6)	123 (56.4)	0.183
Coronary artery disease	3 (33.3)	6 (66.7)	0.645
drinking	41 (41.4)	58 (58.6)	0.904
aspirin	4 (33.3)	8 (66.7)	0.768
β-blockers	8 (38.1)	13 (61.9)	0.795
ACEI/ARB	22 (34.4)	42 (65.6)	0.264
TC (mmol/l)	4.40 ± 1.01	4.56 ± 1.13	0.179
TG (*M* (*P25, P75*), mmoL/L)	1.76 (0.97,2.10)	2.16 (1.12,2.35)	0.058
HDL (mmol/l)	1.07 (0.86,1.23)	0.97 (0.80,1.11)	0.002**
LDL (mmol/l)	2.73 ± 0.77	2.83 ± 0.87	0.251
LP(a)(M, mg/L)	219.65 (60.57,276.3)	184.56 (65.7,221)	0.288
Non-HDL-C (mmol/l)	3.33 (2.69,3.90)	3.59 (2.75,4.30)	0.057
Non-HDL-C/HDL	3.31 ± 1.22	3.86 ± 1.40	0.000*
Occlusive vessel (%)			
Multiple vessels lesions	30 (62.5)	18 (37.5)	0.001*
LAD	39 (38.2)	63 (61.8)	0.552
LCX	21 (28.4)	53 (71.6)	0.015**
RCA	54 (41.9)	75 (58.1)	0.757

CCC, coronary collateral circulation; ACEI, angiotension converting enzyme inhibitors;ARB, angiotensin receptor blocker; TC, total cholesterol; TG, triglyceride; HDL, high-density lipoprotein; LDL, low-density lipoprotein; non-HDL-C, non–high-density lipoprotein cholesterol; TC/HDL, total cholesterol /high-density lipoprotein cholesterol ratio; Non-HDL-C/HDL, non–high-density lipoprotein cholesterol /high-density lipoprotein cholesterol ratio; LAD, left anterior descending artery; LCX, left circumflex artery; RCA, right coronary artery

*P < 0.01, **P < 0.05

### Relationships between non-HDL-C/HDL ratio and Rentrop grade

**Fig. 1:**
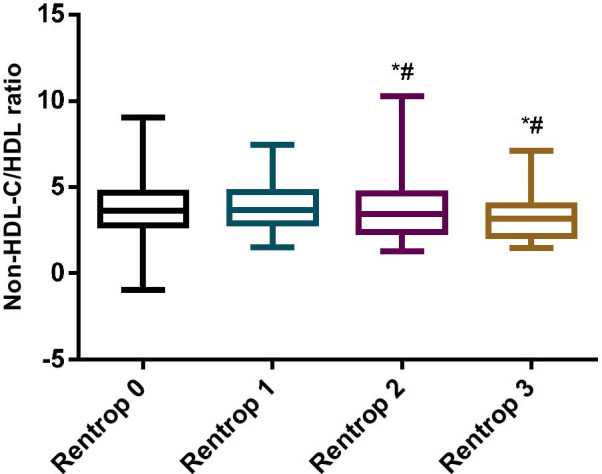
Non-HDL-C/HDL ratio value of patients in each Rentrop grade. *Note*:*P < 0.05 (compared with Rentrop 0 grade). ^#^*P* < 0.05 (compared with Rentrop 1 grade)

Spearman correlation analysis was performed on the non-HDL-C/HDL ratios with the Rentrop grade. Negatively correlated results were obtained between the non-HDL-C/HDL ratio and Rentrop grade (r = − 0.115, *P* = 0.030). The non-HDL-C/HDL ratio in Rentrop 3 (3.14 ± 1.18)was significantly lower than Rentrop 0 ( 3.85 ± 1.41) (*P* = 0.001) and Rentrop 1 ( 3.90 ± 1.36) (*P* = 0.004), The non-HDL-C/HDL ratio in Rentrop 2 (3.41 ± 1.24) was also significantly lower than Rentrop 0 (*P* = 0.012) and Rentrop 1 (*P* = 0.036). The ratio of non-HDL-C/HDL between Rentrop 0 and Rentrop 1, Rentrop 2 and Rentrop 3 showed no significant differences. (*P* > 0.05) (Fig. 1).

### Analysis of risk factors for coronary collateral circulation

The baseline variables that are considered clinically relevant or showed a univariate relationship with the results were used in the multivariate model to evaluate the potential predictors of CCC. The good or poor formation of CCC was used as dependent variable, and candidate factors with a *P* value < 0.2 in univariate analysis (Table 1) were included in the multivariable analysis. The results showed that multiple vessel lesions and non-HDL-C/HDL were independent risk factors for CCC, non-HDL-C/HDL (OR = 1.195, 95%CI = 1.020–1.400, P = 0.027) was significantly associated with the risk of CCC poor formation, the contribution to the risk of poor CCC formation is nearly 1.2 times with per one unit increase in non-HDL-C/HDL (Table 2).

**Table 2:** Multivariable logistic regression analysis of independent factors for CCC

Variables	B	Wald	*P* value	OR	95% CI
Age	− 0.666	0.506	0.908	0.999	0.979–1.019
Non-HDL-C/HDL	0.178	4.879	0.027**	1.195	1.020–1.400
Hypertension	0.195	1.619	0.203	1.343	0.853–2.115
Multiple vessels lesions	0.983	8.943	0.003**	2.671	1.403–5.088
LCX	− 0.671	5.154	0.023**	0.511	0.286–0.912

Non-HDL-C/HDL, non–high-density lipoprotein cholesterol /high-density lipoprotein cholesterol ratio; LCX, left circumflex artery

*P < 0.01, **P < 0.05

### Non-HDL-C/HDL prediction of CCC poor formation with ROC curves

**Fig. 2:**
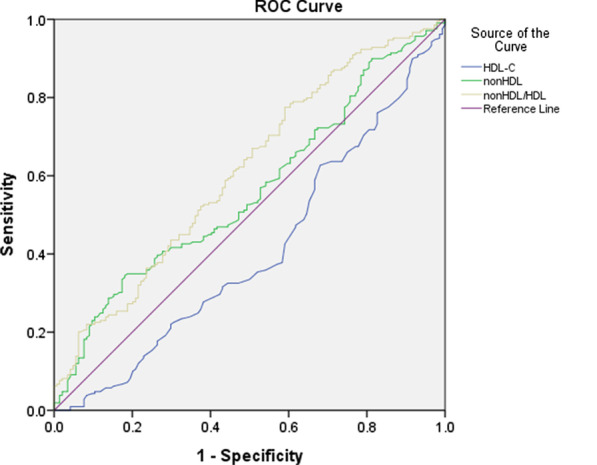
Receiver operating characteristic (ROC) analysis of the relationship between the non-HDL-C/HDL-C ratio and the poor formation of CCC

The ROC analysis revealed that the area under the curve(AUC) of non-HDL-C and HDL for detecting CCC poor formation were 0.560 (95% CI: 0.500–0.620, *P* = 0.056) and 0.405 (95% CI: 0.344–0.465, *P* = 0.002), respectively. However, AUC of the non-HDL-C/HDL ratio for detecting CCC poor formation was 0.611 (95% CI: 0.551–0.671, *P* = 0.000), the expected non-HDL-C/HDL ratio was better than non-HDL-C or HDL for predicting CCC poor formation. The optimal cut-off point was 2.77 (Youden index 0.185), with 77.5% sensitivity and 41% specificity (Fig. 2).

### Study strengths and limitations

The present study demonstrated that in CTO patients 1) the non-HDL-C/HDL ratio in the CCC poor formation group was significantly elevated compared to the CCC good formation group and 2) the non-HDL-C/HDL ratio negatively correlated with the formation of CCC and may be developed as an independent predictor of CCC formation. These results indicated that the non-HDL-C/HDL ratio may be used as a noninvasive biomarker for evaluating CCC in CTO patients, and it will be more feasible and economical.

The study has some limitations. First, the assessment of CCC was achieved only using coronary angiography, which is far less accurate than other methods (e.g., CFI). Second, this study was a small retrospective study, and the influence of selection bias cannot be excluded. Finally, Rentrop grade was defined according to the results of coronary angiography, catheter size and the spatial resolution of angiographic systems could influence the results in this study.

## Discussion

CCC exerts protective effects in CTO patients, such as reducing the occurrence of small myocardial infarction, improving ventricular function, decreasing cardiovascular events and increasing long-term survival rate [6, 12, 13]. The formation of CCC is a complicated process, and several factors contribute to its development. The severity of coronary obstruction and duration of ischemic symptoms positively correlate with poor collateral development [14, 15]. However, patients with diabetes [16], hypercholesterolemia [17], hypertension [18] and hyperuricemia [19] are less likely to develop collateral vessels. However, vascular endothelial growth factor (VEGF) and fibroblast growth factor (FGF) effectively promoted the formation of CCC in animal experiments [20]. The presence and function of CCC were also influenced by plasma chemokine concentrations, such as TNF-α [21], the angiogenic ligand and interferon [22]. Many studies showed that the development of CCC was a pathophysiological process involving multiple mechanisms, including oxidative stress, systematic hypoxia, inflammation and vascular endothelial function [23, 24].

Dyslipidemia is a contributing factor for CAD. Hyperlipidemia reduces vascular endothelial function and inhibits CCC formation [25]. However, different components in blood lipids have different impacts on endothelial function. Non–high-density lipoprotein cholesterol includes all atherogenic cholesterol in the blood, and the most important component of which is LDL-C because increased LDL-C causes damage to vascular endothelial function[26]. The incidence of long-term major adverse cardiovascular events (MACE) increased with increasing levels of non-HDL-C after acute myocardial infarction compared to lowering LDL-C to target [27]. Therefore, the lowering of non-HDL-C is a goal of treatment in the process of anti-atherosclerosis [28]. HDL-C exerts a protective role in the pathophysiological process of atherosclerosis formation and development [29], in which HDL-C plays anti-inflammatory and anti-oxidative roles and protects vascular endothelial cells by carried anti-oxidant enzymes such as paraoxonase-1(PON1), lecithin cholesterol acyltransferase(LCAT), and platelet-activating factor acetylhydrolase (PAF-AH). In particular, PON1 has been suggested to mainly mediate the potential anti-atherogenic capacity of HDL [30–32]. The above research suggested that HDL-C and LDL-C played pivotal roles in the physical function of endothelial cells, and endothelial cells are truly involved in the formation of CCC. HDL-C and LDL-C may affect the functioning of endothelial cells, the formation and function of CCC.

Some studies indicated that lipoprotein ratios were better predictors of CAD than parameters of conventional lipids [9]. Our previous study showed that non-HDL-L/apolipoprotein A-I was highly correlated with the severity of coronary artery lesions and served as a biomarker in the assessment of CAD [33]. Based on the mechanisms and roles of non-HDL-C and HDL-C in the process of atherosclerosis, non-HDL-C/HDL ratio may be an important indicator and predictor of lipid-related disease. Wang et al. reported that the non-HDL-C/HDL ratio was associated with increased non-alcoholic steatohepatitis [34]. Several studies also reported that the non-HDL-C/HDL ratio was an independent factor of insulin resistance, metabolic syndrome and chronic kidney disease [35]. Some studies examined the role of the non-HDL-C/HDL ratio in postmenopausal women, and it could be used to predict the risk of CAD because the ratio was higher in females with carotid atherosclerotic plaque [36, 37]. However, there are few studies focusing on the relationship between this ratio and CCC formation.

The present study used the Rentrop grade to assess the formation of CCC. A negative correlation between the non-HDL-C/HDL ratio and Rentrop grade was found. CCC formation became poorer with increases in the non-HDL-C/HDL ratio. A multivariate logistic regression model showed that the non-HDL-C/HDL ratio was an independent risk factor for the formation of CCC. ROC results indicated that the optimal cut-off value of this ratio was 2.77 for detecting poor formation of CCC, the AUC (0.611) is not very impressive,somehow it has a relatively high sensitivity (77.5%), comparing with non-HDL-C or HDL respectively, non-HDL-C/HDL ratio was better for predicting CCC poor formation. In clinical practice, non-HDL/HDL ratio is easy to obtain and more economical than other methods (e.g. CFI, contrast echocardiography). The degree of coronary artery stenosis was tightly related to CCC formation because it promotes the initiation and is the main determinant of the CCC formation. After the formation of CCC is initiated, the function of endothelial cells could affect the function of CCC. Serum HDL-C and LDL-C levels affect vascular endothelial function. The non-HDL-C/HDL ratio was negatively related to CCC formation in the present study. It may be speculated that these two lipid particles affect endothelial cell function. In conclusion, our study provides a new index to assess and predict the formation of CCC.

The present study provides a theoretical basis for the choosing of lipid-lowering drugs for treating CAD. Lipid-lowering therapy should not only emphasize the reduction of non-HDL-C but also focus on increasing the level of HDL-C. The treatment of CAD should consider the formation and function of CCC.

## Conclusion

The non-HDL-C/HDL ratio in the CCC poorly formed group of CTO patients was significantly elevated compared to the CCC well-formed group. Non-HDL-C/HDL negatively correlated with CCC formation,it independently served as a better predictive factor of CCC formation than non-HDL-C or HDL alone, which may be used as a biomarker for the evaluation of CCC.

## Data Availability

All data generated or analyzed during this study are included in this published article.

## Abbreviations

Non-HDL-C/HDL: Non-high-density lipoprotein cholesterol/high-density lipoprotein cholesterol ratio
CCC: Coronary collateral circulation
CTO: Chronic total occlusive disease
ROC: Receiver operating characteristic
CTOs: Chronic total occlusions
CAD: Coronary artery disease
LDL-C: Low-density lipoprotein cholesterol
non-HDL-C: Nonhigh-density lipoprotein cholesterol
TG: Triglyceride
HDL-C: High-density lipoprotein cholesterol
CI: Confidence interval
